# Native Valve Infective Endocarditis Caused by *Staphylococcus condimenti*: First Case Reported

**DOI:** 10.1016/j.jaccas.2026.109108

**Published:** 2026-06-27

**Authors:** Antonio Terrón, Eduardo Aparicio-Minguijón, Raúl Recio, José Enrique Ruiz-Cabello, Laura Domínguez-Pérez, Juan Meca-Aguirrezabalaga, Francisco López-Medrano

**Affiliations:** a Department of Internal Medicine, University Hospital ‘12 de Octubre’, Madrid, Spain; b Department of Internal Medicine, Pontevedra University Hospital Complex (CHOP), Pontevedra, Spain; c Unit of Infectious Diseases, University Hospital ‘12 de Octubre’, Madrid, Spain; d Department of Microbiology, University Hospital ‘12 de Octubre’, Madrid, Spain; e Department of Nephrology, University Hospital ‘12 de Octubre’, Madrid, Spain; f Department of Cardiology, University Hospital ‘12 de Octubre’, Madrid, Spain; g Department of Cardiac Surgery, University Hospital ‘12 de Octubre’, Madrid, Spain; h Department of Medicine, Instituto de Investigación Hospital 12 de Octubre (imas12), Universidad Complutense, Madrid, Spain; i Centro de Investigación Biomédica en Red de Enfermedades Infecciosas (CIBERINFEC), Instituto de Salud Carlos III (ISCIII), Madrid, Spain

**Keywords:** coagulase-negative staphylococci, infective endocarditis, native valve, *Staphylococcus condimenti*

## Abstract

**Background:**

*Staphylococcus condimenti* is a coagulase-negative staphylococcus previously considered nonpathogenic. We report to our knowledge the first documented case of native-valve infective endocarditis caused by this pathogen.

**Case Summary:**

A 67-year-old man with rheumatic heart disease presented with fever and purpura. Initial IgA-dominant glomerulonephritis and leukocytoclastic vasculitis suggested systemic vasculitis. However, echocardiography revealed a large mitral vegetation. *Scondimenti* was identified by MALDI-TOF mass spectrometry and 16S rRNA sequencing from blood cultures and was subsequently confirmed by resected valve culture. After urgent mitral valve replacement and targeted cefazolin, the patient achieved full recovery and resolution of all immune-mediated complications.

**Discussion:**

This report identifies *Scondimenti* as an emerging pathogen capable of significant valvular destruction and immune-mediated phenomena mimicking systemic vasculitis.

**Take-Home Messages:**

Rare coagulase-negative staphylococci require integrating clinical context and species-level molecular identification to avoid dismissal as contaminants. Emerging pathogens can present with misleading systemic immunological manifestations, requiring high clinical suspicion for infective endocarditis.

## Visual Summary

**Figure:**
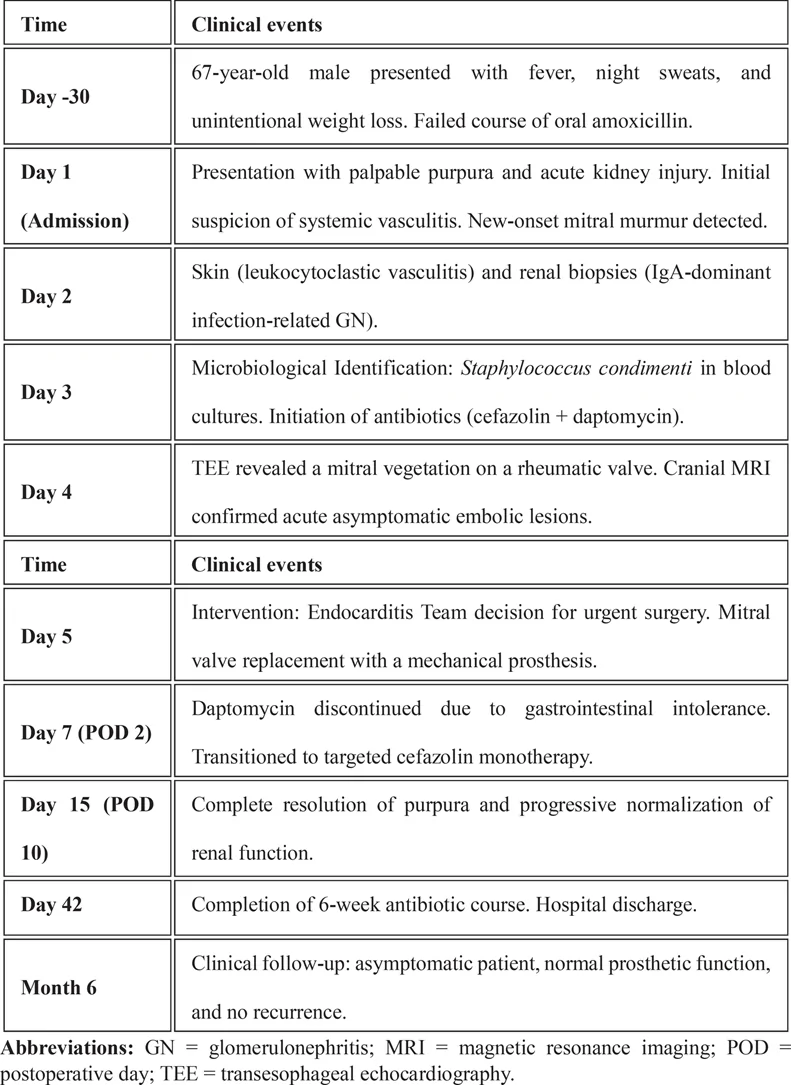

## History of Presentation

A 67-year-old man presented with a 1-month history of fever, night sweats, asthenia, and unintentional weight loss. He had recently completed an unsuccessful course of oral amoxicillin and was being treated by a dermatologist for a cutaneous eruption. On physical examination, the patient was hemodynamically stable. Dermatological assessment revealed palpable purpura symmetrically distributed over the thighs (Figure 1). Cardiac auscultation demonstrated a new-onset mitral murmur, distinct from known baseline findings. There were no signs of congestive heart failure or other peripheral stigmata of infective endocarditis (IE).

**Take-Home Messages**

• Rare species of coagulase-negative staphylococci isolated from blood cultures require integration of clinical context and precise molecular identification to avoid their misinterpretation as contaminants, especially in patients with pre-existing valvular disease.
• Emerging endocardial pathogens can present with misleading systemic immunological manifestations.
• A high index of clinical suspicion and early surgical source control allow for potential recovery from these complications, particularly when dealing with less fulminant microorganisms.

**Figure 1:**
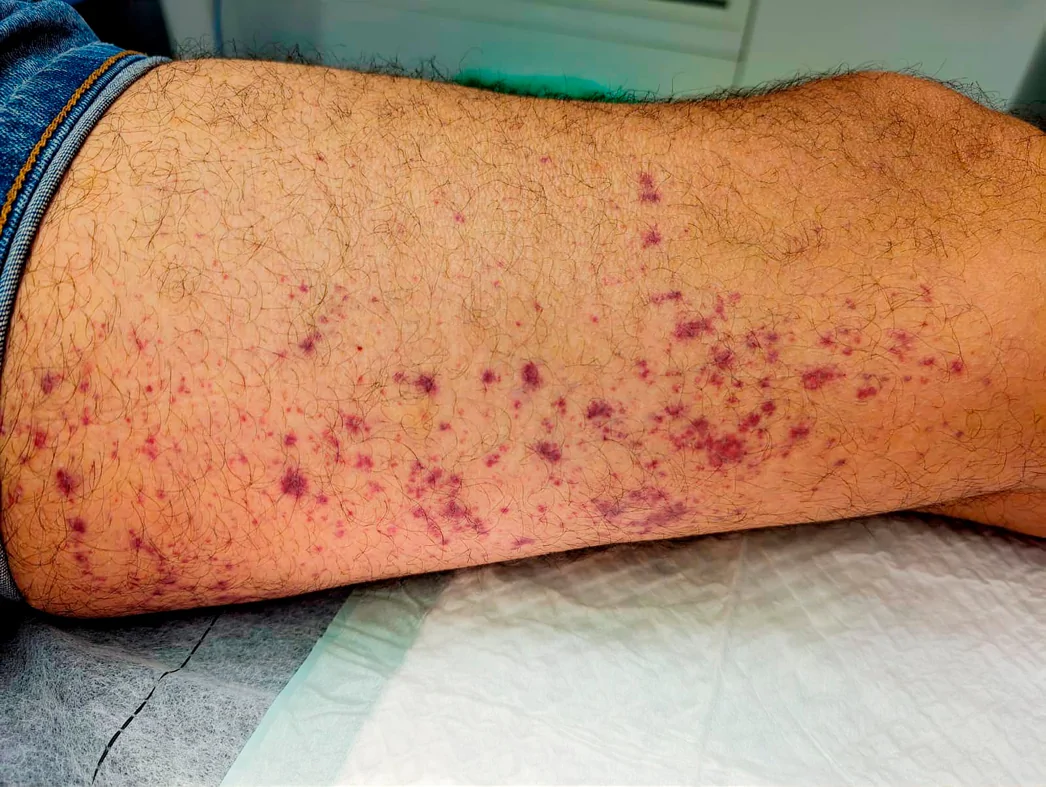
Cutaneous Manifestations This figure illustrates the initial clinical presentation of the patient. Physical examination revealed multiple, nonblanching, erythematous-to-violaceous papules and plaques symmetrically distributed over the thighs. These findings were consistent with palpable purpura secondary to small vessel vasculitis. This clinical sign was pivotal in the initial diagnostic reasoning, directing the work-up toward systemic immune-mediated processes before the diagnosis of subacute infective endocarditis was established.

## Past Medical History

The patient's medical history was significant for mixed rheumatic mitral valve disease and 2 prior ischemic strokes (2 years and 2 months before admission). The most recent stroke involved hospitalization with peripheral venous catheterization and prompted a switch from antiplatelet therapy to apixaban because of suspected cardioembolism. He denied intravenous drug use, recent dental procedures, or prior cardiac surgery.

## Differential Diagnosis

Given the triad of fever, palpable purpura, and acute renal impairment (proteinuria and active urinary sediment), the initial differential diagnosis focused on primary systemic vasculitis, specifically antineutrophil cytoplasmic antibody (ANCA)–associated vasculitis or IgA-vasculitis (Henoch-Schönlein purpura). However, the background of rheumatic heart disease and recent venous catheterization necessitated the exclusion of IE with secondary immune-mediated phenomena.

## Investigations

Laboratory evaluation evidenced a marked systemic inflammatory response (C-reactive protein: 12 mg/dL, erythrocyte sedimentation rate: 51 mm/h) and hyperferritinemia (1,400 ng/mL). Renal function was compromised (peak serum creatinine: 2.5 mg/dL), accompanied by subnephrotic range proteinuria and active urinary sediment. Additionally, N-terminal pro–B-type natriuretic peptide was significantly elevated (3,120 pg/mL).

Regarding the immunological work-up, the comprehensive vasculitis panel—including ANCA and anti–glomerular basement membrane antibodies—and cryoglobulins were negative. However, mild hypocomplementemia was detected, characterized by low C3 levels (75 mg/dL) with preserved C4.

Histopathology confirmed the nature of the end-organ damage: Skin biopsy documented leukocytoclastic vasculitis, while the renal biopsy (Figure 2) revealed IgA-dominant infection-related glomerulonephritis (IRGN). This specific pathological pattern along with the hypocomplementemia was interpreted as immune complex–mediated sequelae of IE, ruling out ANCA-associated vasculitis.

**Figure 2:**
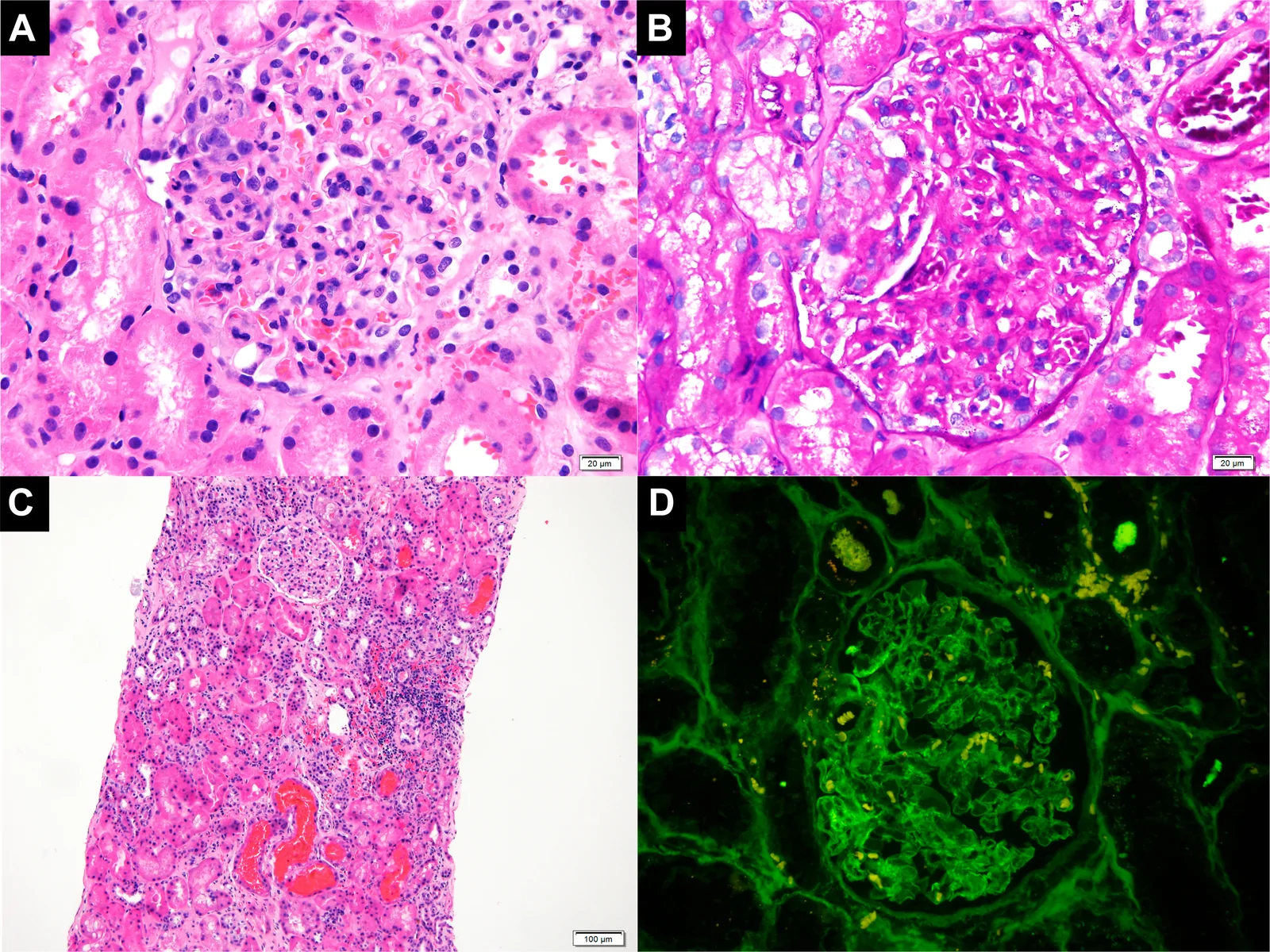
Renal Histopathology Findings Histological evaluation was performed to characterize the patient's acute kidney injury. (A and B) Light microscopy (H&E and PAS stains) revealed a proliferative glomerulonephritis with diffuse endocapillary hypercellularity and segmental necrotizing lesions. (C) The tubulointerstitial compartment (H&E) exhibited a mild mononuclear inflammatory infiltrate, evidence of acute tubular injury, and intratubular red blood cell casts. (D) Immunofluorescence microscopy demonstrated dominant, granular mesangial deposits of polyclonal IgA (2+). Weaker staining for C3 and C1q was also noted (not depicted). These findings confirmed IgA-dominant infection-related glomerulonephritis. H&E = hematoxylin and eosin; PAS = periodic acid-Schiff.

Transesophageal echocardiography visualized a large (22 × 6 × 8 mm), mobile, polylobulated vegetation adherent to the anterior mitral leaflet (Videos 1 to 4), superimposed on progressive rheumatic mitral stenosis (Figure 3). Cranial angio–magnetic resonance imaging confirmed acute cardioembolic ischemic lesions in the carotid and vertebrobasilar territories, while whole-body computed tomography documented splenomegaly and hepatomegaly.

**Figure 3:**
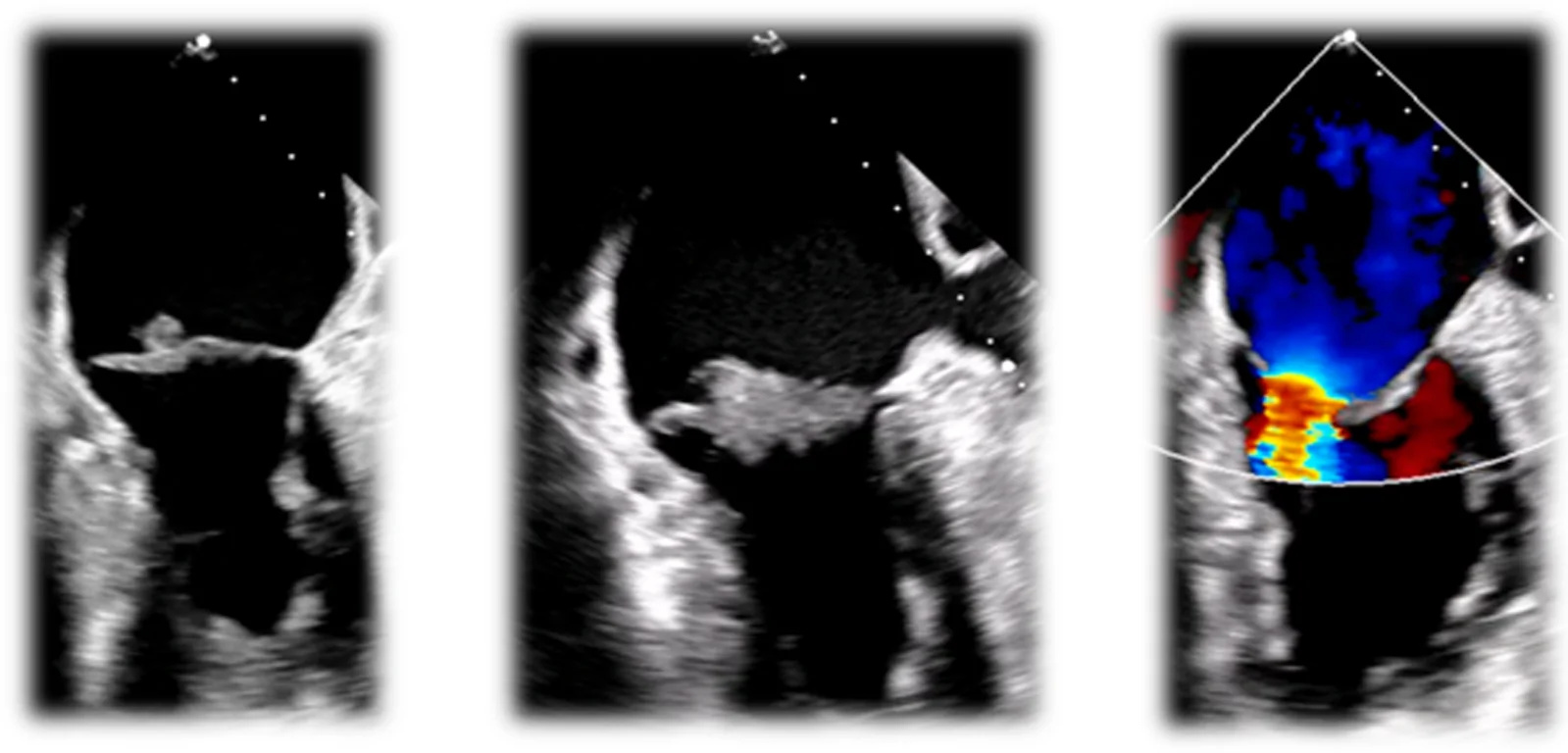
Transesophageal Echocardiography Findings Transesophageal echocardiography was used to assess valvular structure and function. The images demonstrate anterior mitral leaflet doming and commissural fusion consistent with rheumatic involvement. A mobile, friable, and polylobulated vegetation (measuring 22 × 6 × 8 mm) was visualized, anchored to the atrial surface of the anterior mitral leaflet at the A2-A3 level. These results confirmed the diagnosis of infective endocarditis superimposed on rheumatic mitral disease. No perivalvular extension was observed, facilitating the planning of urgent surgical intervention.

The etiological diagnosis was confirmed by the identification of *S condimenti*. Persistent bacteremia was demonstrated in 3 consecutive blood culture sets drawn within a 48-hour period (Figure 4), with bottles flagging positive after 11 hours (BacT/Alert Virtuo, BioMérieux). Identification of the blood isolates was achieved via matrix-assisted laser desorption ionization–time of flight mass spectrometry (MALDI-TOF MS, Bruker Biotyper, score 2.28) and confirmed by 16S rRNA gene sequencing (99% identity, GenBank accession No. PX547893). The etiological role of *S* *condimenti* was finally confirmed by the positive culture of the resected valvular tissue, with species identification verified by MALDI-TOF MS.

**Figure 4:**
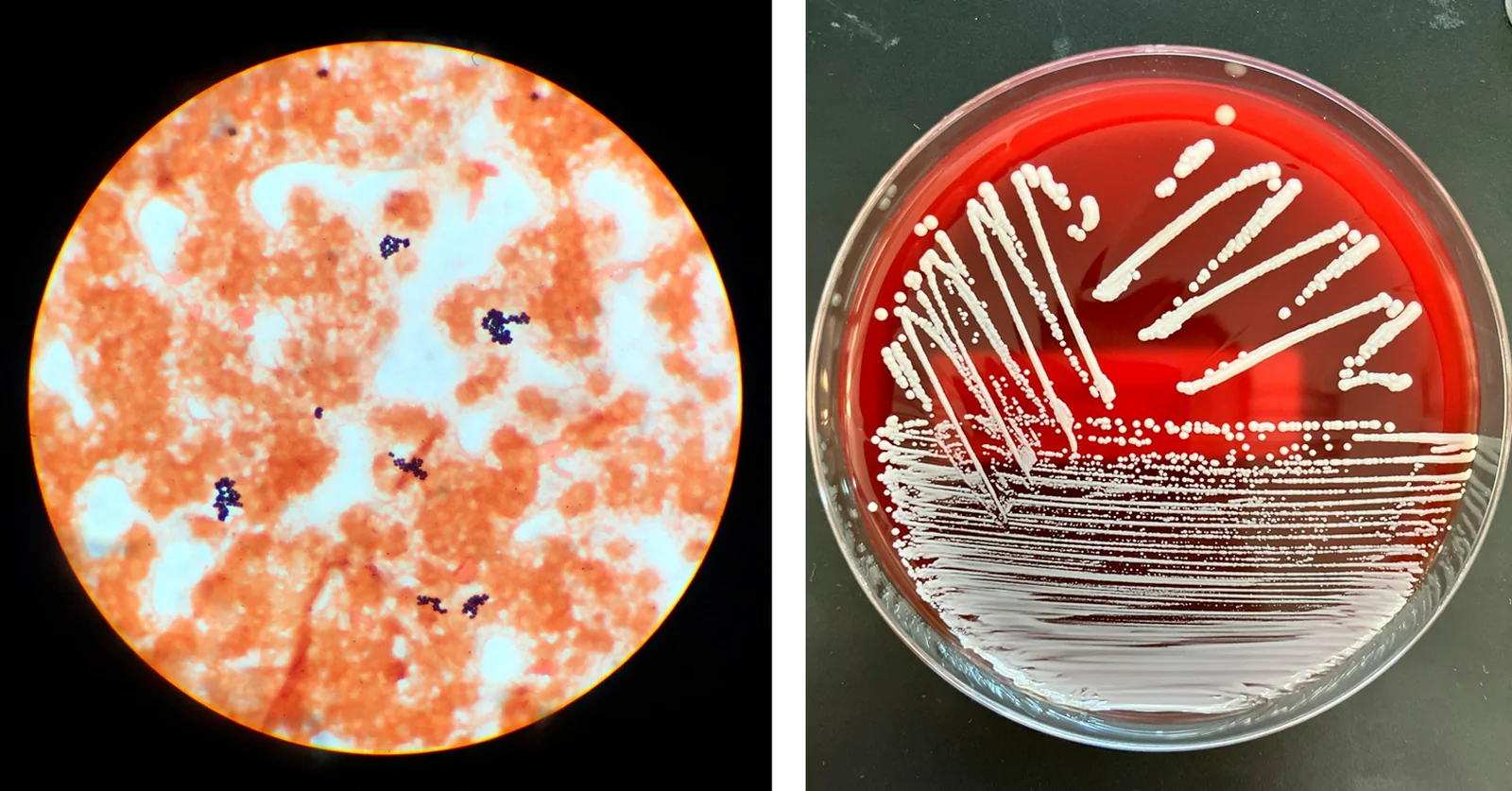
Microbiological Identification Laboratory investigations were performed to identify the causative pathogen. (Left) Gram stain showing Gram-positive cocci in clusters. (Right) At 18 hours, subcultures on blood and chocolate agar under aerobic and 5% CO_2_ atmospheres revealed nonhemolytic catalase-positive white colonies. The use of MALDI-TOF MS and 16S rRNA sequencing allowed for the definitive identification of *Staphylococcus condimenti*. MALDI-TOF MS = matrix-assisted laser desorption ionization–time of flight mass spectrometry.

Susceptibility testing based on EUCAST v.15 breakpoints revealed minimum inhibitory concentrations consistent with a susceptible profile for all tested agents, including penicillin (≤0.06 mg/L), oxacillin (≤0.25 mg/L), levofloxacin (≤1 mg/L), clindamycin (≤0.12 mg/L), vancomycin (0.5 mg/L), and linezolid (≤1 mg/L), guiding definitive therapy.

## Management

Management followed a multidisciplinary endocarditis team approach. Empiric therapy commenced with intravenous cefazolin (2 g every 8 hours) and daptomycin (10 mg/kg every 24 hours). Daptomycin was discontinued on day 7 because of gastrointestinal intolerance (nausea and vomiting). Upon microbiological confirmation of oxacillin-susceptible *S* *condimenti*, treatment was streamlined to targeted cefazolin monotherapy.

Urgent surgical intervention was indicated given high embolic risk, large vegetation size (>10 mm), and progression of valvular stenosis. Intraoperative inspection confirmed a degenerated rheumatic valve with a massive vegetation adherent to the anterior leaflet. The valve was resected (Figure 5), preserving the posterior leaflet, and was replaced with a bileaflet mechanical prosthesis. The procedure was performed under standard cardiopulmonary bypass, with no intraoperative complications.

**Figure 5:**
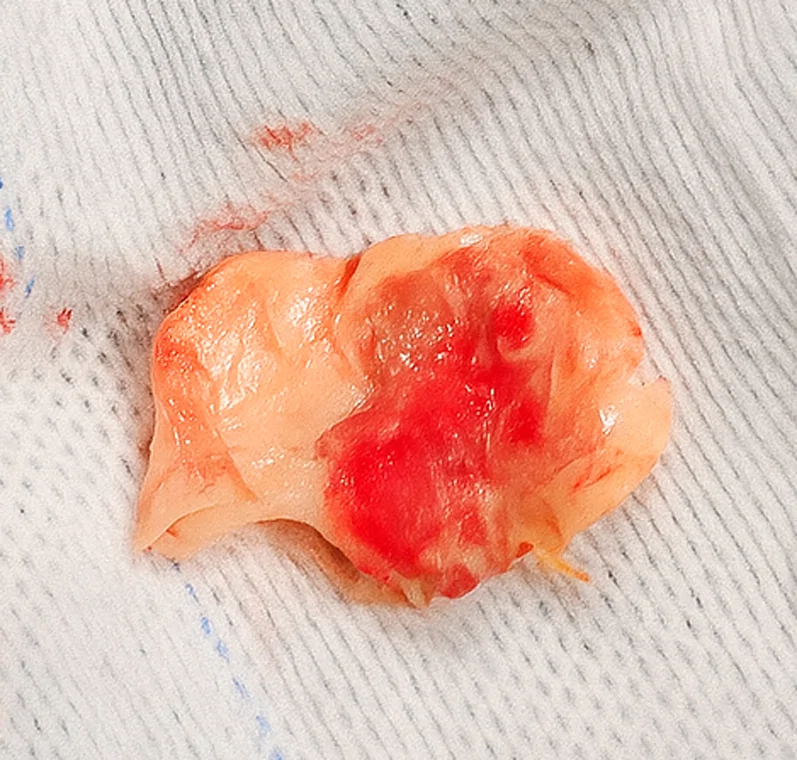
Intraoperative Findings The image shows the native mitral valve after surgical removal, exhibiting marked structural disorganization and a large, adherent vegetation on the anterior leaflet. These findings correlate with echocardiographic measurements and explain the severe valvular dysfunction.

## Outcome and Follow-Up

The postoperative course was uneventful. The patient remained afebrile, with rapid normalization of inflammatory markers and complete resolution of both the cutaneous vasculitis and renal dysfunction. No further embolic events occurred. Predischarge echocardiography verified the normofunction of the mechanical prosthesis.

The patient was transitioned to oral anticoagulation with a vitamin K antagonist. Given the positive valve culture, he completed a full 6-week course of intravenous cefazolin after surgery. At the 6-month follow-up, the patient remained asymptomatic, with no evidence of clinical or microbiological recurrence.

## Discussion

This report represents to our knowledge the first documented case of native-valve IE caused by *S* *condimenti*. Although coagulase-negative staphylococci (CoNS) are traditionally associated with prosthetic valve infections,^1^ this report illustrates the capacity of *S* *condimenti* to cause severe, destructive endocarditis on a native valve.

First described in 1998 in the context of soy sauce fermentation, *S* *condimenti* has been traditionally regarded as an environmental organism of low virulence.^2^ However, recent literature has documented its emergence as an opportunistic human pathogen causing bacteremia, soft tissue infections, meningitis, and spondylodiscitis,^3^, ^4^, ^5^ frequently featuring a nosocomial or health care–associated acquisition.^3^

Still, distinct from the fulminant presentation typical of *S* *aureus*, our case exhibited a subacute course dominated by “classic” vascular and immunological phenomena, allowing for the development of a massive vegetation without early heart failure. This presentation resonates with recent findings from the GAMES cohort,^6^ which characterize native valve CoNS endocarditis as a complex entity where indolent progression can paradoxically lead to severe structural damage.

In our patient, the underlying rheumatic valve disease likely provided the necessary substrate for bacterial adherence and colonization. The progression to a large vegetation and valvular destruction is supported by genomic data identifying putative virulence factors in *S* *condimenti*, including surface adhesins mediating colonization and immune evasion proteins likely triggering immune-complex phenomena.^4^ This challenges the notion that non-*lugdunensis* CoNS are benign in native valve settings.

A pivotal aspect of this case was the misleading clinical presentation, dominated by cutaneous vasculitis and renal failure, which initially raised suspicion of systemic vasculitis. However, the renal biopsy demonstrated IgA-dominant IRGN. This entity is well-documented in association with *S* *aureus*, particularly in diabetic patients.^7^ Nonetheless, the combination of IgA-dominant IRGN in a nondiabetic host caused by a low-virulence species such as *S* *condimenti* is highly unusual.

Morphologically, differentiating IgA-dominant IRGN from primary IgA nephropathy or IgA vasculitis is crucial but challenging.^8^ Furthermore, current literature suggests a generally poor prognosis for this condition, with a high risk of progression to end-stage kidney disease.^8^ Despite this, our patient achieved complete renal recovery. This favorable outcome underscores the potential reversibility of the immune-mediated damage when a less fulminant infectious trigger is promptly eradicated through surgical source control.

From a diagnostic perspective, this case reinforces the critical importance of species-level identification for CoNS isolated in blood cultures. Nonetheless, pathogenicity is ultimately driven by the clinical scenario rather than taxonomy alone. Dismissing non-*epidermidis* CoNS as contaminants without clinical correlation can lead to significant delays in diagnosis. Documenting continuous bacteremia and rapid identification via MALDI-TOF MS, confirmed by 16S rRNA sequencing, were determinants for distinguishing this pathogen from common contaminants.^9^ Furthermore, accurate identification and susceptibility testing allowed for antimicrobial stewardship, facilitating a safe de-escalation to cefazolin given its complete susceptibility. This targeted therapy, combined with early surgery indicated by the “endocarditis team,” was fundamental to the favorable outcome, preventing recurrence despite the initial severity.^10^

## Conclusions

This case represents the first documented evidence of native-valve infective endocarditis caused by *S condimenti*. The successful management of this condition compelled an aggressive, combined medical-surgical intervention, culminating in mitral valve replacement and the reversal of severe systemic immune-mediated complications. This report underscores a fundamental clinical lesson: the critical need to remain vigilant regarding unusual coagulase-negative staphylococci in blood cultures, avoiding their dismissal as contaminants without adequate clinical correlation. Ultimately, it highlights the indispensable role of multidisciplinary teams and precise molecular identification in the timely diagnosis and treatment of emerging endocardial pathogens.

## Funding Support and Author Disclosures

This work was supported by the Centro de Investigación Biomédica en Red de Enfermedades Infecciosas (CIBERINFEC), Instituto de Salud Carlos III (ISCIII), Madrid, Spain. The authors have reported that they have no relationships relevant to the contents of this paper to disclose.

## Appendix

Video 1 Transesophageal Echocardiography Midesophageal 2-chamber view showing a large (22-mm), highly mobile, polylobulated vegetation anchored to the atrial surface of the anterior mitral leaflet, exhibiting significant prolapse into the left atrium during systole.

Video 2 Transesophageal Echocardiography Midesophageal long-axis view with color Doppler demonstrating significant mitral regurgitation. The jet is eccentric, secondary to the restrictive motion of the rheumatic leaflets and the interference caused by the voluminous vegetation during valve closure.

Video 3 Three-Dimensional Transesophageal Echocardiography En face “surgeon's view” providing a detailed anatomical perspective of the mitral valve. The vegetation is clearly localized at the A2-A3 segments, highlighting the structural disorganization caused by the underlying rheumatic disease and the superinfected mass.

Video 4 Three-Dimensional Transesophageal Echocardiography This spatial reconstruction illustrates the origin of the regurgitant jet in relation to the vegetation, confirming the functional impact of the endocardial infection on the mitral apparatus.
